# The Role of Vitamin D in Autoimmune Hepatitis

**DOI:** 10.4021/jocmr1505w

**Published:** 2013-10-12

**Authors:** Khanh vinh quoc Luong, Lan Thi Hoang Nguyen

**Affiliations:** a Vietnamese American Medical Research Foundation, Westminster, California, USA

**Keywords:** Calcitriol, Autoimmune hepatitis, Vitamin D

## Abstract

Autoimmune hepatitis is an inflammation of the liver characterized by the presence of peri-portal hepatitis, hypergammaglobulinemia, and the serum autoantibodies. The disease is classified into 2 distinct types according to the nature of auto-antibodies. Disturbances of the calcium-parathyroid hormone-vitamin D axis are frequently associated with chronic liver disease. Patients with AIH have a high prevalence of vitamin D deficiency. Genetic studies have provided the opportunity to determine which proteins link vitamin D to AIH pathology, namely, the major histocompatibility complex class II molecules, vitamin D receptors, toll-like receptors, cytotoxic T lymphocyte antigen-4, cytochrome P450 CYP2D6, regulatory T cells (Tregs) and the forkhead/winged helix transcription factor 3. Vitamin D also exerts its effect on AIH through non-genomic factors, namely, mitogen-activated protein kinase signaling pathways, γδT cells, interferon-gamma nitric oxide synthase, and reactive oxygen stress. In conclusion, vitamin D may have a beneficial role in AIH and improves liver function in concanavalin A-induced mouse AIH. Calcitriol is best used for AIH because it is the active form of a vitamin D_3_ metabolite and its receptors are present in sinusoidal endothelial cells, Kupffer cells, stellate cells of normal livers, and the biliary cell line.

## Introduction

The auto-immune hepatitis (AIH) is an inflammation of the liver characterized by the presence of peri-portal hepatitis, hypergammaglobulinemia, and serum autoantibodies. The disease is classified into 2 distinct types according to the nature of autoantibodies [1, 2]. Type 1 AIH is characterized by anti-nuclear antibodies and/or smooth muscle antibodies in serum of northern European and American adults. Type 2 AIH is characterized by antibodies to the liver-kidney microsome type 1 (anti-LKM1) and primarily affects children between the ages of 2 and 14 years. Disturbances of the calcium-parathyroid hormone-vitamin D axis are frequently associated with chronic liver disease [3]. Vitamin D deficiency is common in non-cholestatic liver disease and correlates with disease severity [4]. AIH patients have low of vitamin D levels compared with control group [5]. Many studies have shown a significant effect of calcitriol on liver cell physiology. Calcitriol increases intracellular Ca^2+^ in rat hepatocytes [6] and controls DNA polymerase α activity. Calcitriol also controls cytoplasmic and nuclear protein kinase activity and promotes normal liver recovery after partial hepatectomy in rats [7]. Vitamin D has also been shown to have a detoxifying effect in human primary cultured hepatocytes by increasing the expression of P_450_ cytochromes (namely, CYP3A4, CYP2B6, and CYP2C9) [8]. Some studies have failed to detect VDR levels in the liver [9, 10]. However, Gascon-Barre et al [11] demonstrated that human, rat, and mouse hepatocytes express very low nuclear vitamin D receptor (nVDR) mRNA and protein levels. In contrast, the sinusoidal endothelial, Kupffer, and stellate cells of normal livers; the biliary cell line; and rat hepatic neonatal epithelial cells all clearly expressed both nVDR mRNA and protein. Burger et al [12] demonstrated that calcitriol receptors were localized in the nucleus and widely distributed in normal human tissues, including those of the liver, kidney, thyroid, adrenal glands, gastrointestinal tract, breast, and skin. Calcitriol-binding proteins were present in liver nuclei isolated from mice, rabbits, chickens, and cultured rat hepatocytes [13]. A major metabolite of the vitamin D analog 1α-hydroxy-vitamin D_2_, 1α, 24(S)-hydroxy-vitamin D_2_, has been identified in human liver cells in culture and strongly bind to the VDR [14]. Another report demonstrated the presence of VDR mRNA and protein in the livers of rats throughout life [15]. Both in vitro and in vivo models have demonstrated anti-proliferative and anti-fibrotic effects of calcitriol on liver fibrosis [16]. In concanavalin A (ConA)-induced mouse AIH, calcitriol significantly decreased the serum alanine transaminase (ALT) levels and markedly attenuated histological liver damage. The mechanism of action was associated with down-regulation of T-cell-mediated immunity and up-regulation of VDR gene expression [17]. Therefore, we will discuss the role of vitamin D in AIH.

## Genetic Factors Related to Vitamin D and Autoimmune Hepatitis

Studies have suggested that several genes in the major histocompatibility complex (MHC) region promote susceptibility to AIH. Located in the MHC region, human leukocyte antigen (HLA) genes have been implicated in AIH susceptibility. The genes of DRB1*0301 and DRB1*0401 are the susceptibility genes for type 1 AIH in Caucasian American, northern European, and Italian patients [18-20]. The genes of DRB1*1501-DRB5*0101 protect against type 1 AIH in adult Caucasian American [18]. In a systematic review and meta-analysis study in Latin America, DQB1*02, DQB1*0603, DRB1*0405, and DRB1*1301 alleles were found to be risk factors for AIH. However, the DRB1*1302 and DQB1*0301 alleles were protective factors for AIH [21]. Calcitriol is known to stimulate phagocytosis but suppresses MHC class II antigen expression in human mononuclear phagocytes [22, 23].

Genetic studies provide an opportunity to link molecular variations with epidemiological data. DNA sequence variations, such as polymorphisms, exert both modest and subtle biological effect on AIH. Vitamin D exhibits immune-modulatory and anti-proliferative effects through VDR in diseases. Among a variety of VDR gene polymorphisms, BsmI and TaqI have been reported to be associated with autoimmune liver diseases. The FokI polymorphisms of VDR have been linked to AIH in German and Chinese populations [24, 25]. These reports suggest that alteration of VDR function may play a role in AIH.

Toll-like receptors (TLRs) are a group of glycoproteins that function as surface trans-membrane receptors and are involved in the innate immune responses to exogenous pathogenic microorganisms. Substantial evidence exists for an important role of TLRs in the pathogenesis and outcome of AIH. Healthy livers contain low mRNA of TLRs, such as TLR-1, TLR-2, TLR-4, TLR-8, TLR-9, TLR-10, and signaling molecules (MD-2 and My88) compared with other organs [26]. In ConA-induced mouse AIH models, TLRs-My88 signaling accelerates liver damage [27], a CpG-containg oligodeoxynucleotides (CpG-ODN) treatment decrease attenuates ConA-induced mouse AIH [28]. CpG-ODN is a ligand for TLR-9 that can be used as an immunological adjuvant. TLR-3 also has an important role in liver inflammation and injury in ConA-induced mouse AIH models. Treatment with ConA markedly increased TLR-3 expression on liver lymphocytes and sinusoidal endothelial cells, while disruption of the TLR-3 gene abolished ConA-induced liver injury [29]. In AIH, monocytes were higher in number, displayed higher levels of tumor necrosis factor alpha (TNF-α), and expressed higher levels of TLR-4 compared with healthy controls [30]. Calcitriol has been shown to down-regulate intracellular TLR-2, TLR-4 and TLR-9 expression in human monocytes [31]. Interestingly, TLR activation results in the expression of the VDR and 1α-vitamin D hydroxylase in human monocytes [32].

Cytotoxic T lymphocyte antigen-4 (CTLA-4) is involved in the regulation of T cells and upregulated during T-cell-mediated immune response. CTLA-4 functions as a negative regulator by binding to the B7 family co-stimulatory molecules on antigen-presenting cells with higher affinity than CD28. The CTLA-4 protein is known to have a role in the pathogenesis of AIH. Mice vaccinated with a plasmid containing the N-terminal region of mouse CTLA-4 and antigenic region of human CYP2D6 developed anti-LKM1 and anti-LC1 antibodies of the IgG2 subclass. In addition, the ALT levels have been correlated with both the presence of anti-LKM1/anti-LC1 antibodies and liver necroinflammation [33]. The mRNA levels of CTLA-4 were significantly decreased in patients with AIH compared with healthy controls [34]. CTLA-4 polymorphisms are associated with type 1 AIH in northern Caucasian European patients [35], Chinese patients [36], and Argentina children [37]; but not in Brazilian [38], Japanese [39], and German populations [40]. A meta-analysis study demonstrated that CTLA-4 gene +49A/G polymorphisms may be associated with susceptibility to type 1 AIH, while the A/A genotype may be protective against type 1 AIH [41]. Calcitriol promoted regulatory T cell profiles by increasing CTLA-4 and interleukin-10 in mouse colon protein extracts [42]. Calcitriol also stimulated the expression of high levels of CTLA-4 in human CD4^+^ CD25- T cells [43].

The cytochrome P450 (CYP) superfamily of enzymes is responsible for the oxidation, peroxidation, and/or reduction of vitamins, steroids, and xenobiotics, as well as the metabolism of drugs. CYP2D6, an important member of this superfamily, has been identified as the major auto-antigen in type 2 AIH [44]. CYP2D6 is the major target auto-antigen of LKM-1 and is expressed on the plasma membrane of hepatocytes. This finding suggests a pathogenic role for anti-LKM-1 in liver injury [45]. Anti-LKM-1 sera inhibited CYP2D6 activity in vitro but did not inhibit cellular drug metabolism in vivo [46]. Antibodies against CYP2D6 conformational antigenic sites were present in LKM-1-positive sera [47]. The CYP2D6 index was significantly higher in patients with AIH compared with healthy controls [48]. Hintermann et al [49] demonstrated that epitope spreading is initiated at the immune-dominant epitope and later expands to neighboring and remote regions in patients with AIH and the CYP2D6 mouse model. Moreover, CYP2D6 is a potential 25 hydroxyvitamin D-1 α-hydroxylase, which converts vitamin D_3_ into 25OHD, and vitamin D 25-hydroxylase deficiency resulted in vitamin D deficiency [50]. Human 25-hydroxyvitamin D-1 α-hydroxylase mutations also cause vitamin D-dependent rickets type 1 [51].

Regulatory T cells (Tregs) are a distinct lymphocyte with inhibitory properties that affect the activation of the immune system. Tregs are characterized by the expression of CD25^+^ and the forkhead/winged helix transcription factor 3 (Foxp3) [52]. During active disease, CD4^+^CD25^high^ T cells were reduced in numbers, expressed low levels of Foxp3, and were less effective at inhibiting target cell proliferation in patients with AIH compared with healthy controls [53-55]. The Foxp3-positive cells were primarily located in the hepatic lobular peri-sinusoidal spaces and portal tract. The frequency of Tregs was increased in AIH patients compared with control and correlated with the inflammatory activity of the liver [34, 56]. However, vitamin D supplementation was associated with a significantly increased Treg frequency in apparent healthy individuals [57, 58]. Calcitriol can affect human immune responses by regulating Foxp3 expression in CD4^+^ T cells through direct VDR binding to the Foxp3 gene [59]. Calcitriol stimulated the expression of high levels of CTLA-4 and Foxp3 in activated T cells [43]. Taken together, calcitriol may have a role in AIH by inducing Foxp3^+^ regulatory T cells.

Table 1 summarizes the genetic factors associated with vitamin D and autoimmune hepatitis.

**Table 1:** Genetic Factors Related to Vitamin D and Autoimmune Hepatitis (AIH)

Autoimune Hepatitis (AIH)	Vitamin D
**Human Leukocyte Antigen (HLA)**	
DRB1*0301 and DRB1*0401 are the susceptibility genes for type 1 AIH in Caucasian American, northern European, and Italian patients, whereas, DRB1*1501-DRB5*0101 protects against type 1 AIH in adult Caucasian Americans. DRB1*0405 allele is associated with the susceptibility to type 1 AIH in Japanese, Chinese, and Mexican patients. DRB1*1301 is associated with type 1 AIH in Argentine children, Brazilian patients, Venezuelan patients, and Indian patients. Susceptibility to type 2 AIH has been associated DRB1*07 in Brazil, Germany, and Turkish children. In a systematic review and meta-analysis study in Latin America, DQB1*02, DQB1*0603, DRB1*0405, and DRB1*1301 alleles were found to be risk factors for AIH, while DRB1*1302 and DQB1*0301 alleles were protective factors for AIH.	Calcitriol suppresses MHC class II antigen expression in human mononuclear phagocytes and decreases interferon-γ-induced HLA-DR antigen expression in normal and transformed human keratinocytes.
**Vitamin D Receptor (VDR)**	
BsmI and TaqI are reported to be associated with autoimmune liver diseases. The FokI polymorphisms of VDR are linked to AIH German and Chinese populations.	
**Cytotoxic T lymphocyte antigen-4 (CTLA-4)**	
The mRNA levels of CTLAA were significantly decreased in patients with AIH compared with healthy controls. Mice vaccinated with a plasmid containing the N-terminal regional of mouse CTLA-4 and the antigenic region of human CYP2D6 developed anti-LKM1 and anti-LC1 antibodies of IgG2 subclass. In addition, the ALT levels correlated with both the presence of anti-LKM1/anti-LC1 antibodies and presence of liver necroinflammation. CTLA-4 deficiency causes severe lympho-proliferative disorder and multi-organ auto-immunity, leading to massive tissue destruction and early death. CTLA-4 Ig suppressed a lupus-like illness in mouse models and reduced the incidence of diabetes in NOD mice. CTLA-4 polymorphisms are associated with type 1 AIH in northern Caucasian European [53], Chinese patients, and Argentina children; but not in Brazilian, Japanese, German populations. A meta-analysis study demonstrated that CTLA-4 gene +49A/G polymorphisms may be associated with the susceptibility to patients with type 1 AIH, while A/A genotype may be protective against type 1 AIH.	Calcitriol promoted regulatory T cell profiles by increasing CTLA-4 and interleukin-10 in mouse colon protein extracts. Calcitriol also stimulated the expression of high levels of CTLA-4 in human CD4^+^ CD25^-^ T cells.
**Toll-like Receptors (TLRs)**	
In ConA-induced mouse AIH models, TLRs-My88 signaling accelerates the liver damage, whereas a decrease in CpG-containing oligodeoxynucleotides (CpG-ODN) treatment attenuates ConA-induced mouse AIH. CpG-ODN is a ligand for TLR-9 and is used as an immunological adjuvant. LR-3 also has an important role in liver inflammation and jury in ConA-induced mouse AIH models. In AIH, monocytes were higher in number, displayed a higher tumor necrosis factor alpha (TNF-α), and expressed high levels of TLR-4 compared with healthy controls.	Calcitriol has been shown to down-regulate intracellular TLR-2, TLR-4 and TLR-9 expression in human monocytes. TLR activation results in the expression of the VDR and 1α-vitamin D hydroxylase in human monocytes. Biliary epithelial cells show intense immune-reactivity for cathelicidin and VDR.
**Cytochrome P450 Cyp2D6**	
*CYP2D6* is the major target auto-antigen of LKM-1 and expressed on plasma membrane of hepatocytes, suggesting a pathogenic role for anti-LKM-1 in liver injury as a trigger. Anti-LKM-1 sera inhibited *CYP2D6* activity in vitro but did not inhibit cellular drug metabolism in vivo. Antibodies against *CYP2D6* conformational antigenic sites were present in LKM-1-positive sera.	*CYP2D6* is a potential 25 hydroxyvitamin D-1 α-hydroxylase, which converts vitamin D_3_ into 25OHD, and vitamin D 25-hydroxylase deficiency resulted in vitamin D deficiency. Human 25-hydroxyvitamin D-1 α-hydroxylase mutations also cause vitamin D-dependent rickets type 1.
**Regulatory T cells (Tregs) and the forkhead/winged helix transcription factor 3 (Foxp3)**	
In humans, mutations in Foxp3 result in an auto-immune syndrome termed IPEX (immune dysregulation, polyendocrinopathy, enteropathy, X-linked syndrome), an X-linked immuno-deficiency syndrome characterized by insulin-dependent diabetes, thyroiditis, massive T cell infiltration in multiple organs, and chronic wasting. During active disease, CD4^+^CD25^high^ T cells were fewer, expressed low levels of Foxp3, and were less effective at inhibiting target cell proliferation in patients with AIH than healthy controls. The Foxp3-positive cells were primarily located in hepatic lobular peri-sinusoidal spaces and the portal tract. The frequency of Tregs was increased in AIH patients compared with control and correlated with the inflammatory activity of the liver.	Vitamin D supplement was associated with significantly increased Tregs frequency in apparent healthy individuals. Calcitriol can affect human immune responses by regulating Foxp3 expression in CD4^+^ T cells through direct VDR binding to the Foxp3 gene. Calcitriol stimulated expression of high levels of CTLA-4 and Foxp3 in activated T cells.

## The Non-Genetic Role of Vitamin D in Autoimmune Hepatitis

The mitogen-activated protein kinase (MAPK) pathways provide a key link between the membrane-bound receptors that receive these cues and changes in the pattern of gene expression. The MAPK pathways include the extracellular signal-regulated kinase (ERK) cascade, stress activated protein kinases/c-jun N-terminal kinase (SAPK/JNK) cascade, and p38 MAPK/RK/HOG cascade [60]. Activation of p38 MAPK may contribute to the pathogenesis of auto-immune disease via activation of the signal transduction and expression of cytokines and chemokines [61]. The activation of p38 MAPK signaling pathway was up-regulated in experimental AIH, and the inhibition of p38 MAPK reduced hepatic inflammation and injury [62]. IL-17 contributes to the pathogenesis of AIH via induction of MAPK signaling pathway [63]. Apolipoprotein A2 (Apo A2) suppressed ConA-induced hepatitis by inhibiting the phosphorylation of ERK1/2 and cjun and reduced the intra-hepatic infiltration of inflammatory cells [64]. By regulating VDR mRNA expression, the p38 MAPK pathway participates in the mediation of calcium signals and affects lipid accumulation in mouse pre-adipocytes [65]. Zhang et al [66] demonstrated that the up-regulation of MAPK phosphatase 1 by vitamin D inhibited LPS-induced p38 activation and cytokine production in monocytes and macrophages.

T lymphocytes recognize foreign antigen by means of their surface receptor. According to the receptor type, T cells may be divided into αβ and γδ subsets. In peripheral blood, γδT cells represent a small number of T cells. However, γδT cells may play important roles in the pathogenesis of AIH. An elevation in the relative and absolute counts of γδT cells in the peripheral and portal areas of patients with AIH has been detected [67]. γδT cells in AIH patients were more numerous and had higher interferon-gamma (IFN-γ) and granzyme B production than healthy controls [53]. AIH children have an expansion and activation of γδT cells in the peripheral blood [68]. The cytotoxic potential of γδT lymphocytes has been previously demonstrated [69]. In contrast, calcitriol significantly inhibits the pro-inflammatory activity of γδT cells in a dose dependent fashion [70]. The production of calcitriol by macrophages within tuberculous lesions inhibits proliferation and CD44 expression in γδT cells [71].

Most human T cell clones obtained from healthy donors produce IFN-γ, IL-2, IL-4, and IL-5 upon stimulation with various mitogens. Soluble liver antigen-specific IFN-γ responses were significantly more frequent in AIH patients than controls [72]. IFN-γ is positively correlated with transaminase levels and decreases with immune-suppressive treatment or disease remission [73, 74]. Overexpression of IFN-γ-inducible protein 10 was identified in the liver of patients with type 1 AIH [75]. The production of IFN-γ is required for pathogenesis in a mouse model of fulminant liver inflammation and murine model of hepatitis [76, 77]. Additionally, calcitriol decreased serum ALT levels, markedly attenuated the histological liver damage, and caused a reduction of IFN-γ in ConA-induced hepatitis [17]. Calcitriol inhibits CD40-induced IFN-γ and immune-modulatory activity in human monocytes [78] and is a potent suppressor of IFN-γ-mediated macrophage activation [79].

Nitric oxide (NO) is involved in host defense reactions and plays a key role in the pathophysiology of vascular disorders. NO production in both sera and liver tissue increased after liver injury induced by delayed-type hypersensitivity to picryl chloride [80]. Animals pretreated with the inducible NO synthase (iNOS) inhibitor, L-N(6)-(1-iminoethyl)-lysine (L-NIL) had significantly increased serum ALT levels compared with animals challenged with ConA alone. However, pretreatment with NO donor molsidomine dramatically decreased ALT levels in L-NIL-pretreated animals [81]. This result suggests both endogenous and exogenous NO protect liver against CoA-induced liver injury. Increased iNOS expression and nitrotyrosine accumulation was correlated with the histological severity of AIH [82]. Calcitriol-produced by macrophages may provide protection against the oxidative injuries caused by NO burst. Calcitriol is known to inhibit lipopolysaccharides (LPS)-induced immune activation in human endothelial cells [83].

Reactive oxygen species (ROS) have been suggested to play a role in AIH. The amount of ROS found in AIH livers was significantly higher than in healthy human livers [84]. Markers of lipid peroxidation, plasma malondialehyde and 8-isoprostane, were significantly elevated in patients with AIH patients compared with normal controls [85]. The red blood cell glutathione (GSH) concentrations were significantly lower in AIH than normal controls. However, plasma GSH peroxidase activity was significantly higher in AIH patients with elevated ALT values compared with normal controls [86]. In AIH patients, oxidative stress and DNA damage are involved in the bile duct injury similar to primary biliary cirrhosis [87]. Moreover, homologs of NADPH oxidases (NOXs) are major sources of ROS and are expressed in human livers with stage 2-3 AIH [88]. Calcitriol enhances intracellular GSH pools and significantly reduces nitrite production induced by LPS [89]. In rat centrilobular hepatocytes, a vitamin D-deficient diet induced a significant increase in NADPH [90].

Table 2 summarizes the non-genomic role of vitamin D in autoimmune hepatitis.

**Table 2:** Summary of the Non-Genomic Role of Vitamin D in Autoimmune Hepatitis

Autoimmune Hepatitis (AIH)	Vitamin D
**The mitogen-activated protein kinase (MAPK) signaling pathways**	
The activation of p38 MAPK signaling pathway was up-regulated in experimental AIH, and the inhibition of p38 MAPK reduced hepatic inflammation and injury. IL-17 contributes to the pathogenesis of AIH via the induction of MAPK signaling pathway. Apolipoprotein A2 (Apo A2) suppressed ConA-induced hepatitis by inhibiting the phosphorylation of ERK1/2 and cJun and reduced the intra-hepatic infiltration of inflammatory cells.	Up-regulation of MAPK phosphatase 1 by vitamin D inhibited LPS-induced p38 activation and cytokine production in monocytes and macrophages.
**γδT cells**	
An elevation in the relative and absolute counts of γδT cells in peripheral and portal areas of patients with AIH. AIH children are associated with an expansion and activation of γδT cells in the peripheral blood.	Calcitriol significantly inhibits the pro-inflammatory activity of γδT cells in a dose dependent fashion.
**Interferon-gamma (IFN-γ)**	
Soluble liver antigen-specific IFN-γ responses were significantly more frequent in AIH patients. This response was positively correlated with transaminase levels and decreases with immune-suppressive treatment or disease in remission state. Production of IFN-γ is needed for the pathogenesis in a mouse model of fulminant liver inflammation and murine model of hepatitis.	Calcitriol decreased the serum ALT levels and markedly attenuated the histological liver damage, and reduction of IFN-γ in ConA-induced hepatitis. Calcitriol inhibits CD40-induced IFN-γ and immune-modulatory activity in human monocytes and is a potent suppressor of IFN-γ-mediated macrophage activation.
**Reactive Oxygen Species (ROS)**	
The amount of ROS found in AIH livers was significantly higher than in healthy human livers. Markers of lipid peroxidation were significantly elevated in patients with AIH compared with normal controls. Red blood cell glutathione (GSH) concentrations were significantly lower in AIH than normal controls, but plasma GSH peroxidase activity was significantly higher in AIH patients with elevated ALT values compared with normal controls. Homologs of NADPH oxidases (NOXs) are major sources of ROS and was expressed in human livers with stage 2-3 AIH.	Vitamin D may reduce the extent of lipid peroxidation, induces SOD activity in the hepatic antioxidant system in rats, enhances intracellular GSH pools, and significantly reduces nitrite production induced by lipopolysaccharide (LPS). In rat centrilobular hepatocytes, a vitamin D-deficient diet induced a significant increase in NADPH.
**Nitric oxide synthase (NOS)**	
NO production in both serum and liver tissue increased in the liver injury induced by delayed-type hypersensitivity to picryl chloride. Both endogenous and exogenous NO protected liver against CoA-induced liver injury. Increased iNOS expression and nitrotyrosine accumulation correlated with the histological severity of AIH.	Calcitriol-produced by macrophages may provide protection against the oxidative injuries caused by NO burst. Calcitriol is known to inhibit lipopolysaccharides (LPS)-induced immune activation in human endothelial cells.

## Conclusion

The relationship between vitamin D and AIH has been discussed. Vitamin D may have a beneficial role in AIH. Genetic studies have provided the opportunity to determine what proteins link vitamin D to AIH pathology. Vitamin D also exerts its effect on AIH through non-genomic mechanisms. Calcitriol is useful in treating AIH, because it is an active form of a vitamin D3 metabolite, and its receptors are present in the sinusoidal endothelial cells, Kupffer cells, and stellate cells of normal livers, and the biliary cell line.
